# 

**Published:** 1880-05

**Authors:** 


					﻿Whole Number,
CCXXVI.
MAY, 1880.
Number io,
Volume XIX.
THE
BUFFALO
Mddical and Surgical Journal.
EDITORS:
THOS. LOTHROP, M.
P. W. VAN PEYMA, M. D1.,
A. R. DAVIDSON, M.
HERMAN MYNTER, M.
Ll’CIEN HOWE, M. D.
BUFFALO :
Published by the Medical Journal Association.
Read the Notice of this Journal among the Advertisements.
Entered at the Post-Office at Buffalo, N. Y., as Second-Class Mail Matter.
				

